# Type IV laryngo-tracheo-esophageal cleft with CPAM in a preterm twin- a case report

**DOI:** 10.3389/fped.2026.1874038

**Published:** 2026-07-15

**Authors:** Chee Mun Chan, Palaniappan Janaki Abirami, Agnihotri Biswas, Khadijah Binti Abdul Kader

**Affiliations:** Department of Neonatology, Khoo Teck Puat National University Children Medical Institute, National University Hospital, Singapore, Singapore

**Keywords:** CPAM, foregut anomalies, laryngo-tracheal-esophageal cleft, monochorionic and diamniotic twin, prematurity

## Abstract

**Background:**

Laryngo-tracheo-esophageal cleft (LTEC) is a rare congenital anomaly caused by incomplete separation of the foregut. Type IV defects are exceptionally rare and typically carry high mortality and morbidity despite surgical interventions.

**Case presentation:**

We report a preterm neonate born at 34 + 1 weeks’ gestation from a monochorionic diamniotic twin pregnancy. Antenatal ultrasound detected a large microcystic congenital pulmonary airway malformation (CPAM) with mediastinal shift, absent gastric bubble, and suspected esophageal pouch. Immediately upon birth, bedside ultrasound identified the affected twin. Direct laryngoscopy and intubation revealed a common tracheoesophageal lumen; flexible bronchoscopy confirmed a Type IV LTEC with absent posterior tracheal wall extending to the carina and communicating with the oesophagus. Selective intubation of the right bronchus provided temporary ventilation. CT thorax demonstrated a wide anomalous aero-digestive tract, intrathoracic stomach, and large CPAM. Surgical repair was deemed very high risk and disproportionately burdensome due to a composite of factors: severity of the type 4 cleft, associated significant pulmonary and gastrointestinal anomalies, low birth weight, and being a high risk ECMO candidate. Following multidisciplinary counselling, care was redirected to palliation.

**Conclusion:**

This case highlights limitations of fetal imaging, the importance of early bronchoscopy for airway evaluation and stabilization, and the role of palliative care when extensive surgical correction carries risks of significant harm and may not serve in the best interest of the patient.

## Introduction

Laryngo-tracheo-esophageal cleft (LTEC) is a rare congenital anomaly resulting from incomplete separation of the embryonic foregut into distinct respiratory and digestive channels. It encompasses a spectrum of severity ranging from minor interarytenoid defects to complete absence of the posterior laryngotracheal wall extending to the carina. The traditional Benjamin and Inglis classification system categorizes LTEC into four types, with Type IV representing the most extensive and severe form, historically regarded as incompatible with sustained postnatal survival (1). However, recent studies showed limitation of surgical applicability of the traditional Benjamin–Inglis classification, thus subclassification of Type III and IV LTEC better correlates anatomical extent with operative approach, airway management, and procedural risk, thereby providing a more clinically meaningful decision-making framework (2). LTEC accounts for fewer than 0.01% of congenital laryngeal anomalies, although its true incidence is likely underestimated due to diagnostic challenges, early neonatal mortality, and under-reporting (3, 4).

Clinical manifestations correlate closely with anatomical severity. Infants with Types I–II clefts may present later in infancy with chronic aspiration, recurrent lower respiratory tract infections, or subtle aerodigestive symptoms. In contrast, major clefts (Types III–IV) typically present at birth with immediate airway instability, ventilatory failure, and near-universal aspiration (3, 4). Definitive diagnosis relies on direct laryngoscopy and rigid bronchoscopy, which remain the gold standard for delineating cleft extent and differentiating LTEC from phenotypically similar conditions such as tracheal agenesis or severe laryngotracheal stenosis (5).

Embryologically, the larynx, trachea, oesophagus, and proximal lung buds originate from the primitive foregut between the fourth and sixth weeks of gestation. Normal development requires coordinated formation and fusion of the tracheoesophageal septum, along with dorsoventral patterning of the laryngotracheal groove. Disruption of these tightly regulated processes may result in a spectrum of foregut malformations, including LTEC, esophageal atresia, and congenital pulmonary airway malformations (CPAM) (6). This shared developmental origin provides a biological rationale for the frequent coexistence of aerodigestive anomalies and suggests that antenatally detected foregut abnormalities may serve as markers for occult airway pathology (7).

Despite advances in prenatal imaging, antenatal diagnosis of LTEC remains exceptionally challenging. Foetal ultrasonography reliably detects associated thoracic and gastrointestinal abnormalities—such as CPAM, intrathoracic stomach, or esophageal displacement—but offers limited visualization of posterior laryngeal and tracheoesophageal structures (8). Foetal magnetic resonance imaging improves soft tissue contrast and characterization of lung lesions and major airway obstructions, including congenital high airway obstruction syndrome, yet small mucosal or cartilaginous defects such as LTEC frequently remain undetected (9). As a result, many cases are only recognized in childhood during evaluation of chronic respiratory conditions (Type I–II) or during evaluation of respiratory compromise and difficult intubation soon after birth (Type III–IV) (3, 4).

Here, we report a rare case of Type IV LTEC in a preterm monochorionic diamniotic twin with antenatally diagnosed CPAM and intrathoracic stomach. Although significant foregut and thoracic anomalies were identified prenatally, the presence of a catastrophic airway malformation was not anticipated. To our knowledge, reports of Type IV laryngo-tracheo-esophageal clefts from Southeast Asia remain limited. This case affirms the diagnostic challenges associated with antenatal identification of Type IV LTEC, particularly in the presence of concomitant foregut anomalies which should prompt heightened suspicion for associated airway malformations and anticipatory planning for postnatal airway management.

## Case report

A spontaneously conceived, monochorionic diamniotic twin pregnancy was initially booked overseas and transferred to a tertiary referral centre in Singapore at 23 weeks’ gestation. Maternal antenatal screening was unremarkable, with negative infectious serologies and low-risk first-trimester screening. There were no significant maternal risk factors such as advanced maternal age (conceived age: 29 years old), maternal smoking, alcohol consumption or maternal diabetes. There was also no history of illicit drug use during pregnancy or exposure to ionizing radiation. Amniocentesis performed at 18 weeks with whole-exome sequencing revealed no genetic abnormalities.

At 20 weeks’ gestation, antenatal ultrasonography in one twin suggested a congenital diaphragmatic hernia. Subsequent imaging revised this diagnosis to a large microcystic type 3 CPAM occupying the left hemithorax. Serial fetal ultrasonography was performed at approximately 2-week intervals from 23 weeks’ gestation until delivery, with assessments at 23, 25, 27, 29, and 32 weeks’ gestation. At 32 + 5 weeks, the lesion measured 52 × 23 mm, with associated mediastinal shift and absence of a gastric bubble. A presumptive esophageal pouch was noted, raising concern for a foregut malformation rather than isolated pulmonary pathology. Right talipes equinovarus was also identified. Amniotic fluid volume and Doppler studies remained normal. Fetal MRI was not considered for evaluation of suspected CPAM or esophageal atresia.

An antenatal multidisciplinary meeting involving obstetrics, neonatology, genetics, and cardiothoracic surgery teams was convened. The family was counselled regarding the extent and uncertainty around the exact nature of malformations. Possible presentations after birth and treatment strategies were explored including need for radiological investigations, surgical and intensive care management. Four doses of antenatal dexamethasone were administered, in an attempt to reduce the CPAM size (10).

At 34 + 1 weeks’ gestation, an elective caesarean section was performed. Elective delivery at 34 weeks’ gestation was undertaken following optimisation of fetal lung maturity with antenatal corticosteroids, while enabling a controlled multidisciplinary delivery for anticipated airway compromise associated with the foregut anomaly, thereby avoiding the risks of an unpredictable emergency delivery. Immediately after delivery, bedside lung ultrasonography was performed on the first-delivered twin, demonstrating abnormal lung parenchyma consistent with congenital pulmonary airway malformation. Neonatal CPAM typically appears on ultrasound as heterogeneous echogenic lung tissue with cystic or microcystic components and may be associated with mediastinal shift in larger lesions (11). The infant had a birth weight of 1,911 g, and Apgar scores of 7 at 1 min and 9 at 5 min of life. He was apnoeic at birth but responded well to brief bag-mask ventilation and was stabilised on CPAP (PEEP 6 cmH₂O, FiO₂ 0.5), before transfer to the neonatal intensive care unit at 8 min of life.

**Figure 1:**
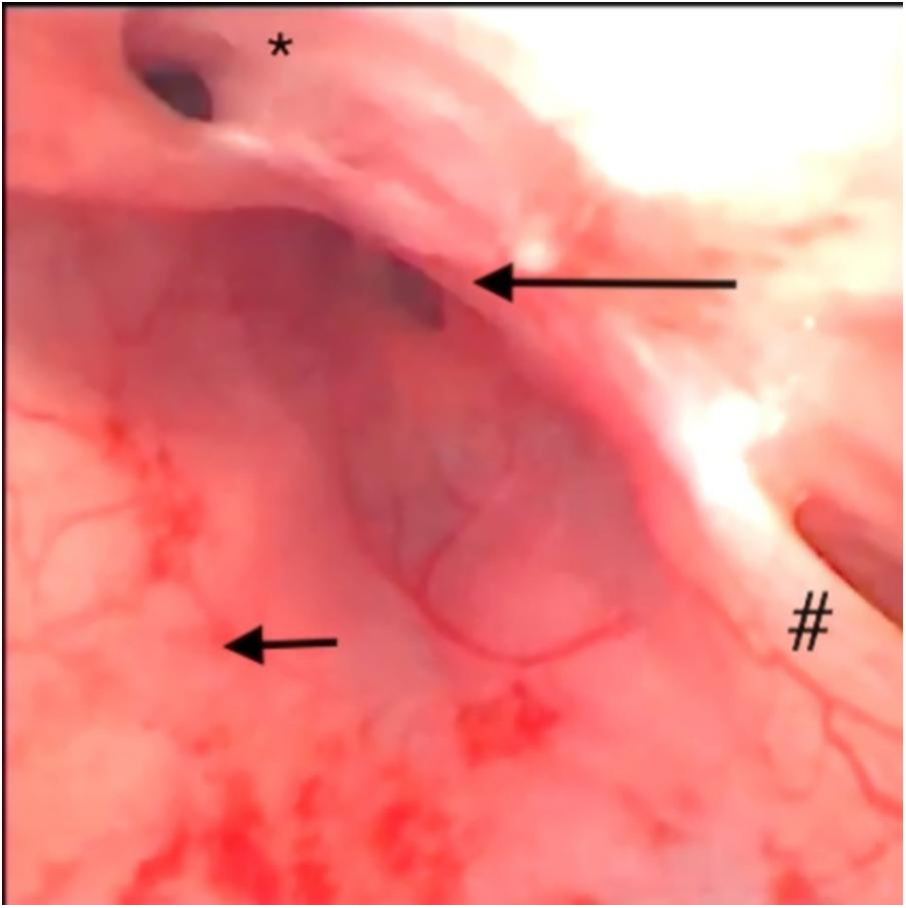
Flexible bronchoscopy image showing Type IV laryngo-tracheo-esophageal cleft. Legends: *-left main bronchus; ^#^- right main bronchus; ^short arrow^- presumptive carina; ^long arrow^- esophageal lumen with rugae.

Elective intubation was attempted due to concerns of esophageal atresia and anticipated respiratory compromise. During video laryngoscopy, a discrete tracheal lumen could not be discerned. A shared tract between the larynx and oesophagus was visualised (Supplementary Video S1). Bedside, flexible bronchoscopy by otorhinolaryngologists confirmed a Type IV laryngo-tracheal-esophageal cleft, characterised by complete absence of the posterior tracheal wall extending to the carina and a single central channel communicating directly with the stomach (Figure 1).

Conventional tracheal intubation was not feasible. A 2.5 mm endotracheal tube was selectively advanced into the right main bronchus to ventilate the unaffected lung. This was technically challenging, as the tube repeatedly entered the common aero-digestive channel. However, under bronchoscopic guidance, selective right mainstem intubation was successfully achieved (Supplementary Video S2). Breathing was supported using conventional patient triggered ventilation.

Given the high risk of aspiration, a Replogle tube was inserted for continuous oropharyngeal and gastric suction. Intravenous antibiotics were commenced in view of risk of aspiration.

**Figure 2:**
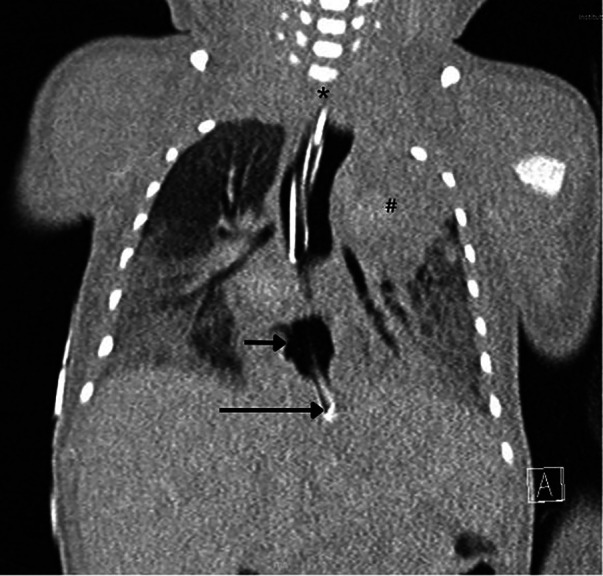
Coronal CTTAP of chest and abdomen showing wide anomalous aero-digestive tract in direct communication with intrathoracic microgastria via hiatal hernia. Left CPAM. Evidence of ETT and OGT in the same shared lumen. Legends: *- common lumen with OGT and ETT; ^#^- Congenital pulmonary airway malformation (CPAM); ^short arrow^- microgastria; ^long arrow^- tip of OGT.

**Figure 3:**
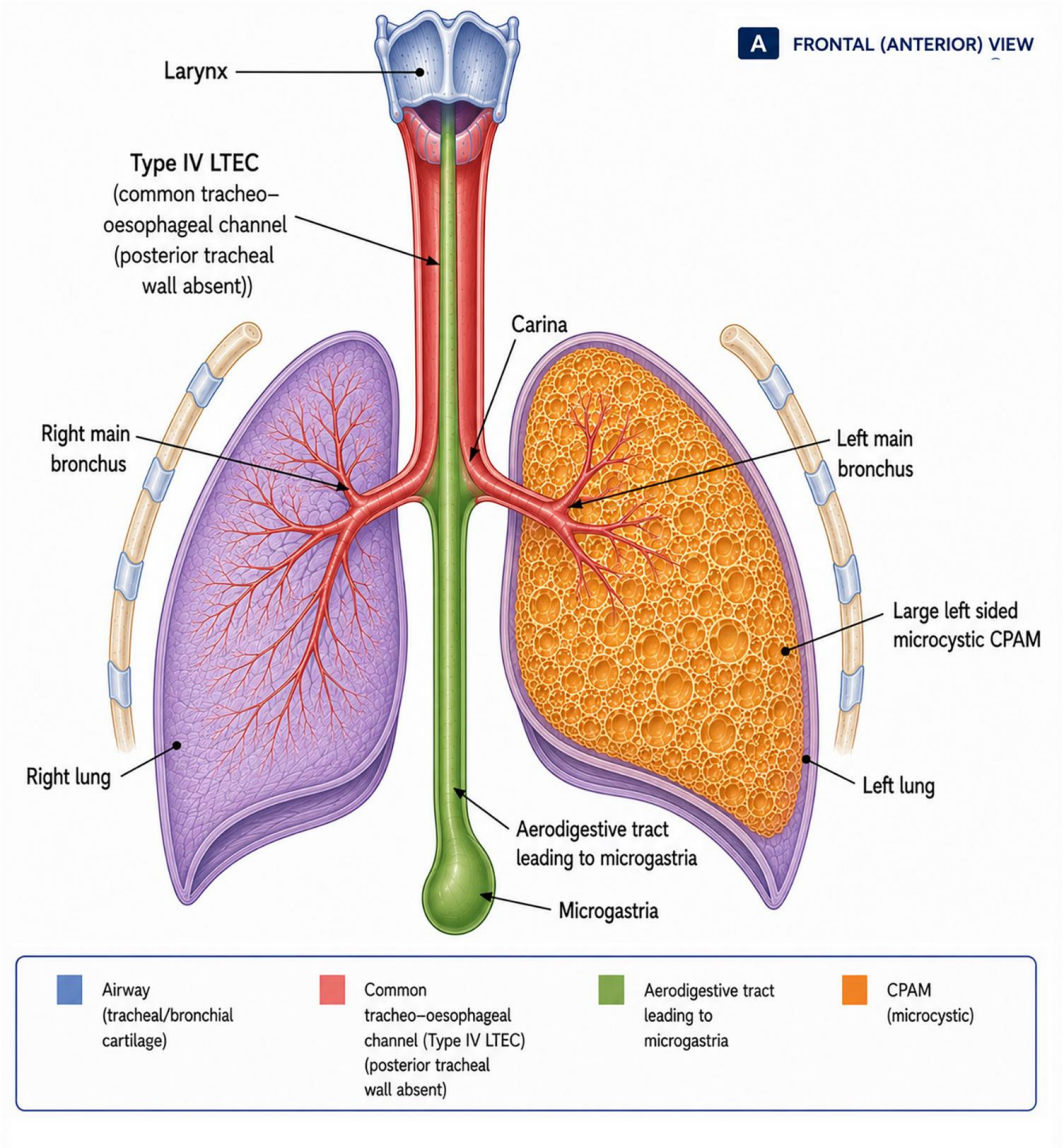
Anatomical schematic of the type IV laryngo-tracheo-esophageal cleft and associated anomalies (frontal view) demonstrating a common esophageal channel extending from the larynx to the carina, consistent with a type IV LTEC, together with a large left microcystic type 3 CPAM and micrograstria.

Computed tomography scan of the thorax, abdomen, and pelvis performed by the second hour of life demonstrated a wide anomalous aero-digestive tract measuring 1.2 × 1.1 cm, complete absence of the posterior tracheal wall, intrathoracic microgastria associated with a hiatal hernia, and absence of an intra-abdominal gastric bubble (Figure 2). The left lung was almost entirely replaced by a large type 3 CPAM (Figure 3). Both endotracheal and orogastric tubes were visualised within the same common channel. These findings were reviewed jointly by neonatology, otorhinolaryngology, paediatric surgery, and cardiothoracic surgery. Surgical repair was deemed infeasible due to anatomical constraints, associated anomalies, and low birth weight.

Following multidisciplinary discussion, the parents elected for redirection of care toward palliation. The infant deteriorated with progressive hypoxaemia and bradycardia, and terminal extubation was performed at 32 h of life. A complete evaluation for associated anomalies was limited by the infant's poor prognosis and short survival. Echocardiography and renal ultrasound were not performed, and therefore the presence of associated congenital cardiac or renal anomalies could not be definitively excluded.

On the other hand, the co-twin was delivered with a birth weight of 1852g. He experienced mild respiratory distress syndrome requiring continuous positive airway pressure support and was successfully weaned to room air by day 5 of life. He demonstrated satisfactory growth and clinical progress and was discharged home on day 25 of life (corrected gestational age 37 + 5 weeks). There was no antenatal evidence of monochorionic twin complications, including twin-to-twin transfusion syndrome (TTTS), selective intrauterine growth restriction (sIUGR), or twin anaemia-polycythaemia sequence (TAPS), during serial fetal surveillance. The twins demonstrated comparable birth weights (1911g and 1852g).

## Discussion

In this pregnancy, antenatal ultrasonography demonstrated a large microcystic congenital pulmonary airway malformation with mediastinal shift, an absent gastric bubble and a suspected esophageal pouch. Laryngo-tracheo-esophageal cleft was not suspected or identified antenatally, reflecting the clinical rarity of this condition and recognised limitations of current prenatal imaging modalities. While foetal ultrasound is highly sensitive for gross thoracic pathology such as CPAM or congenital diaphragmatic hernia, it lacks the spatial resolution required to assess major posterior and deep laryngotracheal structures (12). Foetal magnetic resonance imaging has been shown to provide complementary anatomical information in suspected foregut pathology—particularly improving diagnostic confidence for esophageal atresia through identification of features such as an upper esophageal pouch and refined delineation of mediastinal relationship. However, available evidence suggests that small mucosal or cartilaginous defects, including LTEC, remain difficult to detect even with MRI, and definitive diagnosis typically remains postnatal and via endoscopic evaluation (9). Antenatal “red flags” such as absent gastric bubble or suspected esophageal pouch are therefore non-specific and overlap with more common foregut anomalies, limiting their predictive value for occult airway malformations (8). Similar diagnostic challenges were highlighted by Sonmez et al., who described two Type IV LTEC cases with an antenatal diagnosis of esophageal atresia, emphasizing the limitations of prenatal imaging in distinguishing complex foregut anomalies (13).

Early flexible bronchoscopy was pivotal in this case and should be regarded as the diagnostic cornerstone when neonatal intubation fails in the setting of suspected congenital airway anomaly. Bronchoscopy provides immediate diagnostic clarity, allowing differentiation of LTEC from tracheal agenesis or critical laryngotracheal stenosis and defining the inferior extent of the defect (14). It also offers therapeutic guidance by enabling selective mainstem intubation or optimal tube positioning, facilitating temporary stabilisation, while reducing iatrogenic harm from repeated blind intubation attempts that risk mucosal trauma, airway oedema, hypoxaemia, and aspiration (14, 15).

Definitive management of laryngo-trachea-esophageal cleft depends on defect extent, reconstructable anatomy, and physiological reserve. Type I–II clefts are commonly amenable to endoscopic repair—most often via endoscopic sutured closure or interarytenoid injection augmentation—with favourable outcomes, while selected Type III lesions may be managed either endoscopically or through open transcervical or laryngofissure repair (6, 16). Survival in long-segment Type III–IV laryngo-tracheal-esophageal cleft (LTEC) has improved markedly over the past two decades with advances in airway support and reconstructive techniques. Mathur et al. reported only 44% survival in Type IV LTEC using open cervicothoracic repair with cardiopulmonary bypass, frequently complicated by repair dehiscence and multiple reoperations, whereas Seidl and colleagues demonstrated 89% survival with staged open reconstruction despite persistent long-term morbidity (17, 18). More recently, Tan et al. achieved 86% survival using anterior laryngofissure with posterior cartilage grafting, with no reported recurrence or major postoperative complications, highlighting the evolution toward more durable reconstructive outcomes (19).

Low birth weight further compounds surgical risk. Neonates weighing less than 2,000 g undergoing major thoracic or airway surgery experience higher rates of infection, prolonged ventilation, and mortality, reflecting limited tissue integrity and physiological reserve (17). In this infant, the absence of a posterior tracheal wall extending to the carina, resulting in a single shared airway and esophageal channel, together with a large CPAM leaving minimal functional lung parenchyma, an intrathoracic hypoplastic stomach, and a birth weight of 1,911 g, rendered the risks of surgical intervention disproportionate to any plausible benefit. Although highly specialized reconstructive approaches and ECMO support have been described in selected cases of severe airway anomalies, these options were not considered feasible in our patient given the extensive Type IV cleft anatomy and other hindering factors as above.

Early integration of palliative care is essential when congenital conditions are incompatible with meaningful survival. In catastrophic airway anomalies such as Type IV LTEC, palliative involvement allows care to focus on comfort, dignity, and family-centred support without delaying diagnostic clarification or multidisciplinary discussion (20). Ethical frameworks emphasise non-maleficence, proportionality, and respect for parental autonomy, recognising that redirection of care toward palliation represents active, compassionate treatment rather than abandonment (21).

Most LTEC series originate from North America and Europe, with minimal representation from Southeast Asia. Reporting cases from underrepresented regions broadens global awareness, supports realistic counselling, and contributes to pooled data needed to refine antenatal risk stratification and system preparedness. Documenting non-survivable presentations is as important as reporting successful repairs, as both inform ethical, proportionate care.

## Conclusion

This report describes the one of the few documented cases of Type IV laryngo-tracheo-esophageal cleft from Southeast Asia, highlighting the extreme rarity of this anomaly and the limitations of current antenatal imaging in detecting associated foregut pathology. Current antenatal imaging modalities may fail to identify subtle but catastrophic airway defects such as laryngeal cleft. Therefore, heightened antenatal suspicion, anticipatory airway preparedness, and early bronchoscopy evaluation at birth are therefore essential when complex foregut anomalies are identified.

## Data Availability

The raw data supporting the conclusions of this article will be made available by the authors, without undue reservation.

## Supplementary material

Supplementary Video S1 Video laryngoscopy.

Supplementary Video S2 Flexible bronchoscopy.

## References

[1] Benjamin B Inglis A. Minor congenital laryngeal clefts: diagnosis and classification. Ann Otol Rhinol Laryngol. (1989) 98(6):417–20. 10.1177/000348948909800603 2729823

[2] Jáuregui EJ Propst EJ Johnson K. Current management of type III and IV laryngo-tracheal-esophageal clefts: the case for a revised cleft classification. Curr Opin Otolaryngol Head Neck Surg. (2020) 28(6):435–42. 10.1097/MOO.0000000000000669 33109943 PMC8966410

[3] Johnston DR Watters K Ferrari LR Rahbar R. Laryngeal cleft: evaluation and management. Int J Pediatr Otorhinolaryngol. (2014) 78(6):905–11. 10.1016/j.ijporl.2014.03.015 24735606

[4] Rahbar R Rouillon I Roger G Lin A Nuss RC Denoyelle F. The presentation and management of laryngeal cleft: a 10-year experience. Arch Otolaryngol Head Neck Surg. (2006) 132(12):1335–41. 10.1001/archotol.132.12.1335 17178945

[5] Chien W Ashland J Haver K Hardy SC Curren P Hartnick CJ. Type 1 laryngeal cleft: establishing a functional diagnostic and management algorithm. Int J Pediatr Otorhinolaryngol. (2006) 70(12):2073–9. 10.1016/j.ijporl.2006.07.021 16959329

[6] Ginzel M Huber N Bauer L Kluth D Metzger R. Development of the foregut and the formation of the trachea and esophagus in rat embryos. A symphony of confusion. Front Cell Dev Biol. (2023) 11:1092753. 10.3389/fcell.2023.1092753 36824366 PMC9941168

[7] Kluth D Fiegel H. The embryology of the foregut. Semin Pediatr Surg. (2003) 12(1):3–9. 10.1053/spsu.2003.50003 12520468

[8] Epelman M Kreiger PA Servaes S Victoria T Hellinger JC. Current imaging of prenatally diagnosed congenital lung lesions. Semin Ultrasound CT MR. (2010) 31(2):141–57. 10.1053/j.sult.2010.01.002 20304322

[9] Ethun CG Fallon SC Cassady CI Mehollin-Ray AR Olutoye OO Zamora IJ. Fetal MRI improves diagnostic accuracy in patients referred to a fetal center for suspected esophageal atresia. J Pediatr Surg. (2014) 49(5):712–5. 10.1016/j.jpedsurg.2014.02.053 24851753

[10] Gallagher LT Lyttle BD Dawson-Gore C Vaughn AE Breckenfelder C Reynolds R. The effect of steroids on prenatally diagnosed lung lesions. J Pediatr Surg. (2024) 59(5):969–74. 10.1016/j.jpedsurg.2023.11.004 38042733

[11] Corsini I Parri N Ficial B Dani C. Lung ultrasound in the neonatal intensive care unit: review of the literature and future perspectives. Pediatr Pulmonol. (2020) 55(7):1550–62. 10.1002/ppul.24792 32339409

[12] Spitz L. Oesophageal atresia. Orphanet J Rare Dis. (2007) 2:24. 10.1186/1750-1172-2-24 17498283 PMC1884133

[13] Sonmez K Karabulut R Turkyilmaz Z Turkyilmaz C Isik B Eryilmaz S. Our experience in two cases of type IV laryngo-tracheal-esophageal cleft (LTEC) with a diagnosis of antenatal esophageal atresia. Pan Afr Med J. (2017) 26:55. 10.11604/pamj.2017.26.55.10647 28451032 PMC5398864

[14] Watters K Ferrari L Rahbar R. Laryngeal cleft. Adv Otorhinolaryngol. (2012) 73:95–100. 10.1159/000334452 22472237

[15] Foglia EE Ades A Sawyer T Glass KM Singh N Jung P. Neonatal intubation practice and outcomes: an international registry study. Pediatrics. (2019) 143(1):e20180902. 10.1542/peds.2018-0902 30538147 PMC6317557

[16] Thiel G Clement WA Kubba H. The management of laryngeal clefts. Int J Pediatr Otorhinolaryngol. (2011) 75(12):1525–8. 10.1016/j.ijporl.2011.08.020 21937125

[17] Mathur NN Peek GJ Bailey CM Elliott MJ. Strategies for managing type IV laryngo-tracheal-esophageal clefts at great ormond street hospital for children. Int J Pediatr Otorhinolaryngol. (2006) 70(11):1905–11. 10.1016/j.ijporl.2006.06.009 16901551

[18] Seidl E Huber RM Schweiger C. Long-term outcome of patients with long-segment laryngo-tracheal-esophageal clefts. Pediatr Pulmonol. (2021) 56(6):1405–13. 10.1002/ppul.25133

[19] Tan HK Ong J Loh WS. Outcomes of surgical repair of type III and IV laryngo-tracheal-esophageal clefts with posterior cartilage grafting. Int J Pediatr Otorhinolaryngol. (2024) 180:111900. 10.1016/j.ijporl.2024.111900

[20] Noorily MJ Farmer DL Flake AW. The association of complete laryngo-tracheal-esophageal cleft with left lung agenesis: pathophysiological clues provided by an experiment of nature. J Pediatr Surg. (1998) 33(10):1546–9. 10.1016/s0022-3468(98)90495-x 9802811

[21] Janvier A Farlow B Barrington KJ. Parental hopes, interventions, and survival of neonates with trisomy 13 and trisomy 18. Am J Med Genet C Semin Med Genet. (2016) 172(3):279–87. 10.1002/ajmg.c.31526 27550159

